# Integrated Transcriptomic and Proteomic Analysis Reveals Differential Gene and Protein Expression and Signaling Pathways During a 20 Km Endurance Exercise and Recovery in Mongolian Horses

**DOI:** 10.3390/ani15131981

**Published:** 2025-07-05

**Authors:** Xinzhuang Zhang, Yuanyi Liu, Wei Ma, Lianhao Li, Dongyi Bai, Manglai Dugarjaviin

**Affiliations:** 1 Key Laboratory of Equus Germplasm Innovation, Ministry of Agriculture and Rural Affairs, Hohhot 010018, China; 13470105913@163.com (Y.L.);; 2 Inner Mongolia Key Laboratory of Equine Science Research and Technology Innovation, Inner Mongolia Agricultural University, Hohhot 010018, China; 3 College of Animal Science, Inner Mongolia Agricultural University, Hohhot 010018, China

**Keywords:** Mongolian horses, endurance, exercise, transcriptomic analysis, proteomic analysis, adaptive response

## Abstract

Mongolian horses are renowned for their remarkable endurance and ability to adapt to harsh environments. To delve deeper into the molecular mechanisms that underlie these traits, researchers conducted a comprehensive analysis of transcriptomic and proteomic changes in Mongolian horses at three distinct time points: before, immediately after, and 24 h following a 20 km run. The transcriptomic analysis uncovered significant variations in gene expression patterns across these time points. Specifically, 291 differentially expressed genes (DEGs) were identified when comparing pre-exercise to post-exercise conditions, 832 DEGs in the comparison between post-exercise and 24 h post-exercise, and 127 DEGs in the comparison of pre-exercise to 24 h post-exercise. Notably, key genes involved in metabolic activities and cellular proliferation, such as *PI3K* and *LDHA*, exhibited significant upregulation immediately after exercise but demonstrated a downward trend 24 h post-exercise. Concurrently, the proteomic analysis revealed 49 differentially expressed proteins (DEPs) in the pre-exercise versus post-exercise comparison, 61 DEPs in the post-exercise versus 24 h post-exercise comparison, and 101 DEPs in the pre-exercise versus 24 h post-exercise comparison. Some proteins, like PDK4 and GLUL, remained upregulated at 24 h post-exercise, whereas others, such as PFKM and LDHA, showed signs of recovery or downregulation. By integrating the transcriptomic and proteomic data, we were able to pinpoint overlapping DEGs/DEPs and implicate crucial signaling pathways, including the HIF-1 signaling pathway and glycolysis, in the molecular response of Mongolian horses to exercise. These findings offer insights into the endurance adaptation mechanisms of the Mongolian horse.

## Simple Summary

This study explored the molecular responses of Mongolian horses to a 20 km run by examining transcriptomic and proteomic changes at three time points: before, immediately after, and 24 h post-exercise. Transcriptomic analysis identified numerous differentially expressed genes involved in cell proliferation and energy metabolism, while proteomic analysis revealed differentially expressed proteins with varying expression patterns over time. Integrated analysis highlighted key molecules and pathways mediating the horses’ adaptive response to exercise.

## 1. Introduction

In the field of life sciences, gaining a deep understanding of gene expression and its regulatory mechanisms is central to unveiling the key processes underlying organismal growth and development, metabolic adaptation, disease onset, and progression. Transcriptomics and proteomics, as two pillars of modern biotechnology, provide powerful tools for exploring these complex processes [1].

Transcriptomics, leveraging advanced techniques such as RNA-seq, enables comprehensive and in-depth analysis of gene expression patterns in specific tissues or cells under particular biological conditions. This analysis not only elucidates the intricate relationships between genes and biological traits or disease occurrence but also, through differential expression of transcripts in time and space, provides vital clues for identifying genes involved in cellular metabolic pathways and discovering genes with specific functions [2]. In equine science, transcriptomic techniques are extensively applied to compare muscle gene expression differences among different horse breeds at various developmental stages or training statuses [3,4]. These studies have revealed changes in gene expression related to muscle fiber type transformation, mitochondrial function, oxidative phosphorylation, and muscle structure, offering invaluable insights into our understanding of horse athletic performance, muscle growth mechanisms, and endurance capabilities [5].

The Mongolian horse, as one of the most renowned horse breeds in China and globally, occupies a significant position in the equestrian industry due to its exceptional endurance and unique charm [6]. However, during intense exercise, horses experience oxidative stress, leading to muscle damage and reduced athletic performance. Therefore, elucidating the adaptive mechanisms of horses during exercise is crucial for enhancing the athletic performance of Mongolian horses and safeguarding their health [7]. The preliminary research conducted by Bou et al. [8] has provided us with numerous valuable insights. By employing a series of advanced technologies such as transcriptome sequencing, they initiated a preliminary exploration of the endurance performance of Mongolian horses and successfully revealed that traditional Mongolian endurance training can induce transitions in muscle fibers, as well as changes at the metabolic and transcriptional levels. Moreover, muscle-specific non-coding RNAs may contribute to these transcriptomic changes during the training process. Nevertheless, further research is needed to delve deeper into the metabolic and muscle regulatory mechanisms of Mongolian horses during exercise.

Proteomics, on the other hand, focuses on the study of all proteins in an organism, exploring their interactions, functional relationships, structures, modification states, and expression levels [9]. As the ultimate executors of biological functions, changes in protein expression directly reflect an organism’s response to environmental stress, growth and development processes, and the onset and progression of diseases. In the field of exercise science, proteomic techniques are employed to investigate changes in protein expression under exercise-induced stress, providing new perspectives for understanding exercise-induced fatigue characteristics, muscle damage mechanisms, and the effects of exercise training [10,11,12,13].

However, the correlation between the transcriptome and proteome is not strong, indicating that translation and subsequent regulatory steps play crucial roles in protein expression [14]. Consequently, the combined analysis of transcriptomics and proteomics has emerged as a comprehensive strategy for exploring the relationship between mRNA and protein expression levels. This integrated analysis not only leverages the unique advantages of both omics in revealing gene expression differences but also, through their complementarity, provides a powerful tool for comprehensively understanding gene expression and unveiling complex physiological and pathological processes in organisms [15]. In studies on horse skeletal muscle satellite cells, combined whole-transcriptome and -proteome analyses have revealed the molecular mechanisms by which leucine regulates the proliferation and differentiation of these cells, offering new insights into understanding horse muscle development and metabolic adaptation [16].

In summary, the combined analysis of transcriptomics and proteomics provides a powerful means for gaining a deep understanding of gene expression and its regulatory mechanisms in organisms, as well as for unraveling the athletic performance and adaptive mechanisms of horses. The aim of this study is to present an integrated transcriptomic and proteomic analysis of muscle in Mongolian horses before and after endurance exercise in order to elucidate the molecular mechanisms underlying their endurance adaptation.

## 2. Materials and Methods

### 2.1. Muscle Sample Collection

Four Mongolian horse stallions, aged 24 months ± 1.5 months and all originating from Xilin Gol League, Inner Mongolia Autonomous Region, were enrolled in this study. During the study period, they were managed under a free-grazing system, allowing them to forage freely, and were subjected to a 20 km endurance exercise protocol. Prior to conducting biopsy sampling, the horses were sedated with butorphanol (0.02 mg/kg) and detomidine (0.02 mg/kg). Using a Bergström needle with a 6 mm external diameter, muscle biopsies were performed at a depth of 60 mm. To minimize potential adverse effects on training effectiveness, an initial biopsy was taken from the left gluteus medius two weeks before training. Biopsies were obtained from the right gluteus medius immediately after exercise and again 24 h post-exercise. The samples were split for RNA and protein extraction, preserved in liquid nitrogen immediately after collection, and stored at −80 °C upon return to the laboratory for subsequent transcriptomic sequencing analysis. The Laboratory Animal Welfare and Ethics Committee of Inner Mongolia Agricultural University (No. NND2024009) approved all sampling procedures, which also adhered to regulatory standards.

### 2.2. RNA Extraction from Muscle Tissue and Reverse Transcription

RNA extraction was performed using the Trizol method [17,18]. RNA extraction was carried out by grinding muscle tissue in liquid nitrogen, adding Trizol, and vortexing. After centrifugation, the supernatant was transferred, and chloroform was added, followed by another vortexing and standing period. The upper aqueous phase was then transferred, mixed with pre-cooled isopropanol, and allowed to stand. After centrifugation, the supernatant was discarded, leaving an RNA pellet which was washed with 75% ethanol, centrifuged again, and air-dried. Finally, the RNA was dissolved in sterilized 0.1% DEPC water. The extracted RNA was divided into two portions: one portion was frozen at −80 °C for subsequent RNA sequencing, while the other portion was subjected to reverse transcription.

Subsequently, the extracted mRNA from tissue samples underwent rigorous reverse transcription using the PrimeScript™RT Master Mix (Perfect Real Time) kit from TaKaRa Bio Inc. (Dalian, China) as referenced in Table S1 [19].

### 2.3. Real-Time Quantitative PCR

Primers were designed with the Primer (5.0) software, using referencing sequences from NCBI (https://www.ncbi.nlm.nih.gov, accessed on 10 January 2025), and they were later synthesized by Sangon Biotech Co., Ltd. (Shanghai, China). For real-time quantitative PCR, glyceraldehyde-3-phosphate dehydrogenase (*GAPDH*) was chosen as the internal reference gene, and the procedure was repeated three times for technical replication. The PCR process was conducted with a fluorescent quantitative PCR detection system (BIO-RAD, Hercules, CA, USA). The relative gene expression was determined using the 2^−∆∆Ct^ method [20]. The primer sequence information is shown in Table S2; the reagent composition is shown in Table S3, and the PCR amplification procedure is shown in Table S4.

### 2.4. RNA Transcriptome Sequencing (RNA-Seq)

Based on the Illumina platform, transcriptome sequencing of muscle tissue was conducted. The frozen RNA samples obtained in Section 2.2 was thawed and evaluated for their quality using Nanodrop 2000 spectroscopy (Thermo Fisher Scientific, Waltham, MA, USA), agarose gelelectrophoresis (Biowest, Madrid, Spain), and the Agilent 5300 Fragment Analyzer (Agilent, Santa Clara, CA, USA). The RNA was then reverse transcribed into cDNA using PrimeScriptTM RT Master Mix (TaKaRa Bio Inc., Dalian, China), followed by purification, fragment selection, and PCR amplification to enrich the library. Finally, the sequencing library was quantified using Qubit 4.0 (Thermo Fisher Scientific, Waltham, MA, USA) and sequenced on the Illumina NovaSeq 6000 platform (Illumina, San Diego, CA, USA) after bridge PCR amplification on the cBot to generate clusters [21].

### 2.5. Protein Extraction from Muscle Tissue

For protein extraction, 100 mg of muscle tissue was placed in a 1.5 mL centrifuge tube, and 1 mL of lysis buffer was added. Then, 10 uL of PMSF was added to the tube, and the sample was homogenized using a homogenizer until completely lysed. The sample was centrifuged at 12,000 r for 2 min. The supernatant was collected and aliquoted into 1.5 mL centrifuge tubes [22]. For protein quantification, we utilized the BCA (Bicinchoninic Acid) kit (Solarbio Science & Technology Co., Ltd., Beijing, China).

### 2.6. Proteomics Sequencing

Quantitative analysis was performed using the proteome discovery software, which employs the isobaric labeling method based on unique peptide intensity summation for protein quantification. Protein intensity is calculated by summing the intensities of its matched unique peptides. Following the acquisition of protein quantitative data, normalization was carried out by extracting protein intensities from different samples and applying central transformation to obtain relative quantitative values (R). Differential quantitative analysis was then performed to identify differentially expressed proteins among different sample groups.

### 2.7. Bioinformatics Analysis

#### 2.7.1. Statistics of Raw Sequencing Data

Statistical methods were employed to analyze the base distribution and quality variations across each cycle of all sequencing reads. A quality assessment of the raw sequencing data was conducted for each sample, encompassing the following aspects: (1) statistical analysis of the distribution of the A/T/G/C base content; (2) statistical analysis of the base quality distribution; (3) statistical analysis of the base error rate distribution.

#### 2.7.2. Quality Control of Raw Sequencing Data

Raw sequencing data were filtered to obtain high-quality sequencing data (clean data), ensuring the smooth progression of subsequent analyses. Following quality control, the filtered data were subjected to statistical analysis and quality assessment again. The following software was used: fastp (version: v1.0.1) (https://github.com/OpenGene/fastp, accessed on 10 January 2025).

The filtered clean reads from each sample were aligned to the Equus caballus (horse) reference genome (version: EquCab3.0), which is publicly accessible via the Ensembl database (version: EquCab3.0) (http://www.ensembl.org/Equus_caballus/Info/Index, accessed on 10 January 2025). Alignment was performed using HiSat2 (version: 2.2.1) (http://ccb.jhu.edu/software/hisat2/index.shtml, accessed on 10 January 2025), a widely used bioinformatics tool for short-read mapping. The alignment rates across samples ranged from 95.87% to 96.62%, indicating high-quality mapping of sequencing data to the reference genome. To assess alignment quality, we evaluated metrics including sequencing saturation, gene coverage, and the distribution of reads across genomic regions and chromosomes. These analyses confirmed the robustness of the alignment and provided a foundation for downstream expression quantification and differential expression analysis.

#### 2.7.3. Expression Level Analysis

The expression levels of genes and transcripts were quantitatively analyzed using the software RSEM (version: v1.3.3), and the regulatory mechanisms of the genes were elucidated by integrating sequence functional information. The software used was RSEM (http://deweylab.github.io/RSEM/, accessed on 15 January 2025).

#### 2.7.4. Differential Expression Analysis

After obtaining the read counts of genes, differential expression analysis of genes between samples was conducted for multi-sample (≥2) projects to investigate the functions of differentially expressed genes. The screening criteria for significantly differentially expressed genes (DEGs) were as follows: FDR < 0.05 and |log_2_FC| ≥ 1. The following software was used: DESeq2 (version: 3.21) (http://bioconductor.org/packages/stats/bioc/DESeq2, accessed on 15 January 2025).

#### 2.7.5. GO Annotation and KEGG Enrichment Analysis of Differential Genes

Genes were classified based on the biological processes (BPs) they participate in, the cellular components (CCs) they constitute, and the molecular functions (MFs) they realize, utilizing the GO database. The GO database was used (version: 1.24.4) (http://geneontology.org/, accessed on 16 January 2025).

KEGG PATHWAY enrichment analysis was performed using the Python scipy package (version: 1.14.1), with the computational principle similar to that of GO analysis, employing Fisher’s exact test for calculations. The Benjamini–Hochberg (BH) method was used for multiple testing corrections, and genes with a corrected *p*-value less than 0.05 were defined as significantly enriched in the differentially expressed genes. The software used was as follows: the Python scipy package (https://scipy.org/install/, accessed on 16 January 2025).

#### 2.7.6. Protein–Protein Interaction Network Analysis

Protein–protein interaction (PPI) network analysis is based on the interaction relationships among proteins, utilizing the interaction data from the STRING database to construct a PPI network. This network visualization helps to elucidate the interplay between proteins and identify specific protein clusters and core regulatory proteins that are affected under experimental conditions. The analyses were conducted using the online platform of the Majorbio Cloud Platform (www.majorbio.com) [23].

## 3. Results

### 3.1. Transcriptome Changes in Muscles of Mongolian Horses Pre- and Post-Exercise

#### 3.1.1. Comparative PCA of the Transcriptome Pre- and Post-Exercise

**Figure 1:**
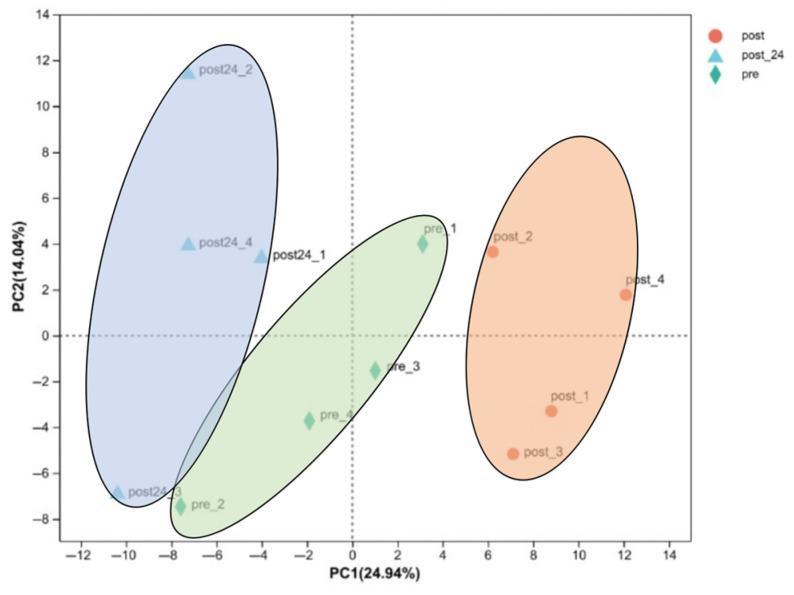
PCA of the transcriptome pre- and post-exercise. The sample points in the figure are marked with different colors and shapes: orange circles represent “post-exercise” samples; blue triangles represent “24 h post-exercise” samples, and green diamonds represent “pre-exercise” samples. The sample points form several distinct clusters: the left cluster is mainly composed of “24 h post-exercise” samples, the middle cluster contains “pre-exercise” samples, and the right cluster is mainly composed of “post-exercise” samples.

In the post-exercise, 24 h post-exercise, and pre-exercise groups, sample clustering was distinct with no significant overlap observed, indicating substantial differences in the overall transcriptomic profiling metrics among the three groups. Samples within each group were relatively clustered (Figure 1).

#### 3.1.2. Differential Gene Expression (DEG) Analysis Pre- and Post-Exercise with Validation by Real-Time Fluorescent Quantitative PCR (RT-qPCR)

**Figure 2:**
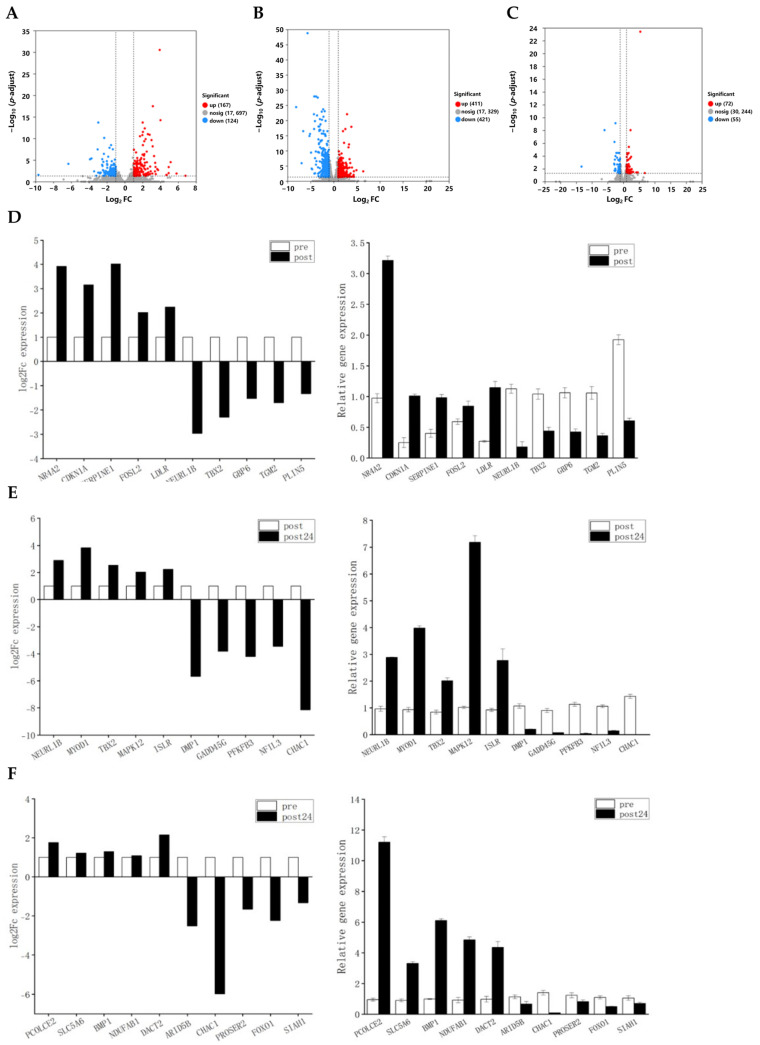
DEGs analysis volcano plot and statistical significance display. (**A**) Volcano plot of expression differences between pre- and post-exercise. Red points indicate significantly upregulated genes; blue points indicate significantly downregulated genes, and gray points indicate non-significant genes. (**B**) Volcano plot of expression differences between post-exercise and 24 h post-exercise. Red points indicate significantly upregulated genes; blue points indicate significantly downregulated genes, and gray points indicate non-significant genes. (**C**) Volcano plot of expression differences between pre-exercise and 24 h post-exercise. Red points indicate significantly upregulated genes; blue points indicate significantly downregulated genes, and gray points indicate non-significant genes. (**D**) Comparison plot of RNA-Seq and RT-qPCR for differentially expressed genes pre- and post-exercise. (**E**) Comparison plot of RNA-Seq and RT-qPCR for differentially expressed genes between post-exercise and 24 h post-exercise. (**F**) Comparison plot of RNA-Seq and RT-qPCR for differentially expressed genes between pre-exercise and 24 h post-exercise.

In the comparison of gene expression between pre-exercise and post-exercise conditions, a total of 291 differentially expressed genes (DEGs) were identified, comprising 167 upregulated and 124 downregulated genes (Figure 2A). In the comparison between post-exercise and 24 h post-exercise conditions, 832 DEGs were identified, including 411 upregulated and 421 downregulated genes (Figure 2B). Furthermore, in the comparison between pre-exercise and 24 h post-exercise conditions, 127 DEGs were identified, consisting of 72 upregulated and 55 downregulated genes (Figure 2C). To validate the accuracy of the transcriptome data, five upregulated and five downregulated DEGs were selected from each of the pairwise comparison groups, and their expression levels were measured using RT-qPCR. The results demonstrated that all genes exhibited consistent trends between RNA-Seq and RT-qPCR measurements (Figure 2D–F), thereby confirming the reliability of the RNA-Seq data.

#### 3.1.3. GO and KEGG Enrichment Analysis of DEGs

**Figure 3:**
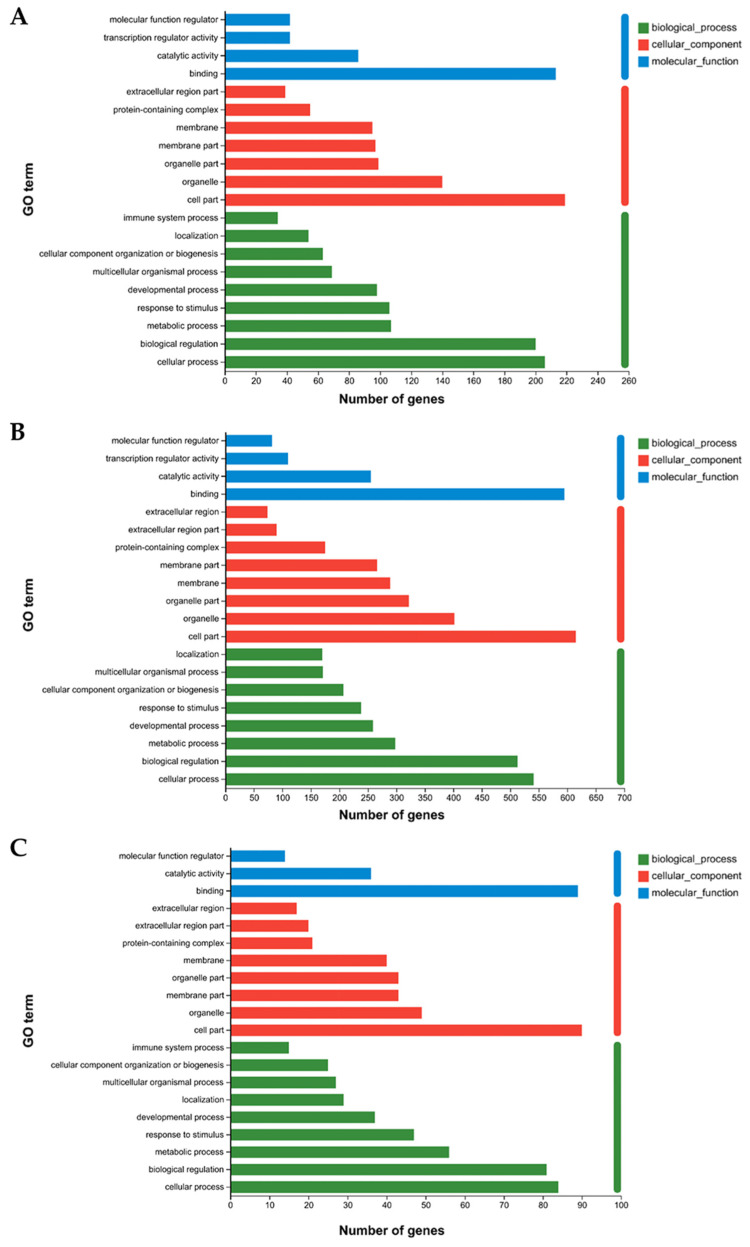

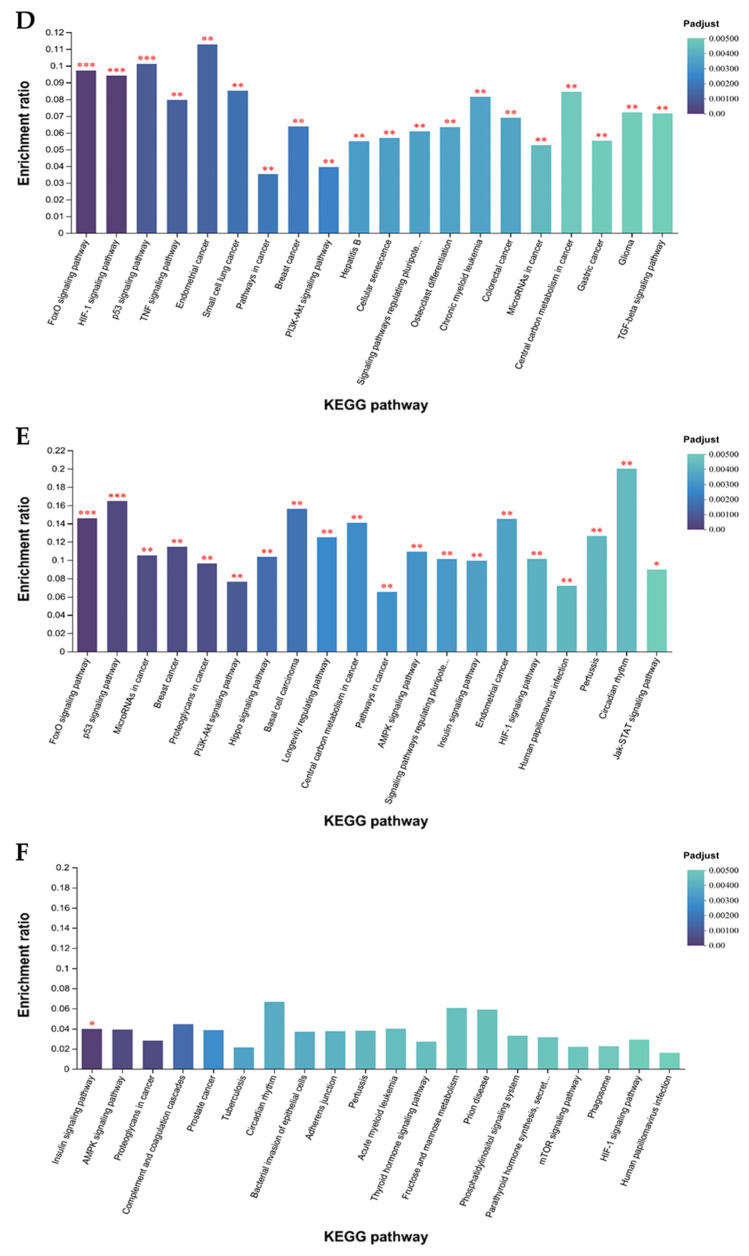
GO and KEGG enrichment analysis of DEGs. (**A**) Numbers of genes falling within enriched GO terms pre- and post-exercise. (**B**) Numbers of genes falling within enriched GO terms post-exercise and 24 h post-exercise. (**C**) Numbers of genes falling within enriched GO terms pre- and 24 h post-exercise. (**D**) Plot of KEGG enrichment analysis comparing pre- and post-exercise. *** denotes *p*-adjust < 0.001, ** denotes *p*-adjust < 0.01. (**E**) Plot of KEGG enrichment analysis comparing post-exercise and 24 h post-exercise. *** denotes *p*-adjust < 0.001, ** denotes *p*-adjust < 0.01, and * denotes *p*-adjust < 0.05. (**F**) Plot of KEGG enrichment analysis comparing pre- and 24 h post-exercise. * denotes *p*-adjust < 0.05.

Gene Ontology (GO) functional annotation was performed on the differentially expressed genes (DEGs). When comparing the pre-exercise state with the post-exercise state (Figure 3A), the DEGs were significantly enriched for terms such as binding, cellular process, biological regulation, and organelle. In the comparison between the post-exercise state and the state 24 h post-exercise (Figure 3B), the DEGs were notably enriched for terms related to binding function, cellular process, biological regulation, and organelle. For the comparison between the pre-exercise state and the state 24 h post-exercise (Figure 3C), the DEGs were significantly enriched for terms including cellular component, binding function, cellular process, and biological regulation.

Kyoto Encyclopedia of Genes and Genomes (KEGG) enrichment analysis was conducted on the DEGs. In the comparison between the pre-exercise and post-exercise states (Figure 3D), the DEGs were significantly enriched for signaling pathways such as the FOXO signaling pathway, the HIF-1 signaling pathway, the p53 signaling pathway, the TNF signaling pathway, the PI3K-Akt signaling pathway, cellular senescence, signaling pathways regulating the pluripotency of stem cells, and the TGF-beta signaling pathway. When comparing the post-exercise state with the state 24 h post-exercise (Figure 3E), the DEGs were significantly enriched in the FoxO signaling pathway, the p53 signaling pathway, the PI3K-Akt signaling pathway, the AMPK signaling pathway, signaling pathways regulating the pluripotency of stem cells, the insulin signaling pathway, the HIF-1 signaling pathway, and the Jak-STAT signaling pathway. In the comparison between the pre-exercise state and the state 24 h post-exercise (Figure 3F), the results indicated that only one signaling pathway, namely the insulin signaling pathway, was enriched.

### 3.2. Proteome Changes in Muscles of Mongolian Horses Pre- and Post-Exercise

#### 3.2.1. Comparative PCA of the Proteome Pre- and Post-Exercise

**Figure 4:**
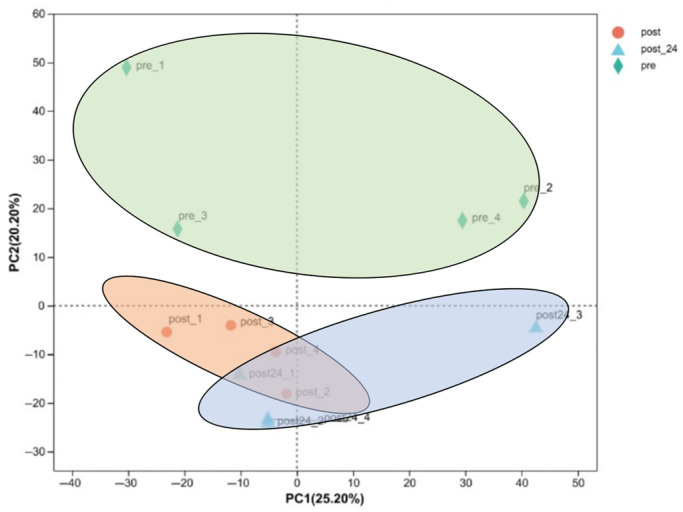
PCA of the proteome pre- and post-exercise. The sample points in the graph are represented by different colors and shapes: orange circles represent “post-exercise” samples; blue triangles represent “24 h post-exercise” samples, and green diamonds represent “pre-exercise” samples. The sample points form several distinct clusters: the cluster on the left is mainly composed of “24 h post-exercise” samples; the cluster in the middle contains “pre-exercise” samples, and the cluster on the right is mainly composed of “post-exercise” samples.

Distinct clustering patterns were observed among the samples in the post-exercise, 24 h post-exercise, and pre-exercise groups. Notably, no significant overlap was detected between the post-exercise and 24 h post-exercise groups compared to the pre-exercise group. However, a moderate degree of overlap was observed between the post-exercise and 24 h post-exercise groups (Figure 4).

#### 3.2.2. Differential Protein Expression (DEP) Analysis Pre- and Post-Exercise

**Figure 5:**
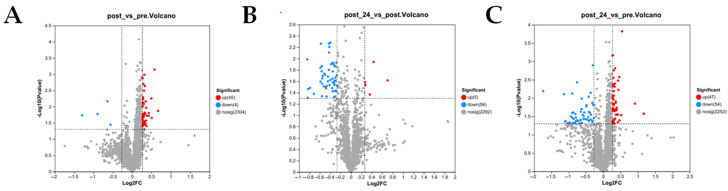
DEP analysis volcano plot. (**A**) Volcano plot of expression differences between pre-exercise and post-exercise. Red points indicate significantly upregulated proteins; blue points indicate significantly downregulated proteins, and gray points indicate non-significant proteins. (**B**) Volcano plot of expression differences between post-exercise and 24 h post-exercise. Red points indicate significantly upregulated proteins; blue points indicate significantly downregulated proteins, and gray points indicate non-significant proteins. (**C**) Volcano plot of expression differences between pre-exercise and 24 h post-exercise. Red points indicate significantly upregulated proteins, blue points indicate significantly downregulated proteins, and gray points indicate non-significant proteins.

In the statistical analysis of protein expression between post-exercise and pre-exercise conditions, a total of 49 significantly differentially expressed proteins were identified, including 45 upregulated and 4 downregulated proteins (Figure 5A). In the statistical analysis of protein expression between 24 h post-exercise and post-exercise conditions, a total of 61 significantly differentially expressed proteins were identified, including 5 upregulated and 56 downregulated proteins (Figure 5B). In the statistical analysis of protein expression between 24 h post-exercise and pre-exercise conditions, a total of 101 significantly differentially expressed proteins were identified, including 47 upregulated and 54 downregulated proteins (Figure 5C).

#### 3.2.3. GO and KEGG Enrichment Analysis of DEPs

**Figure 6:**
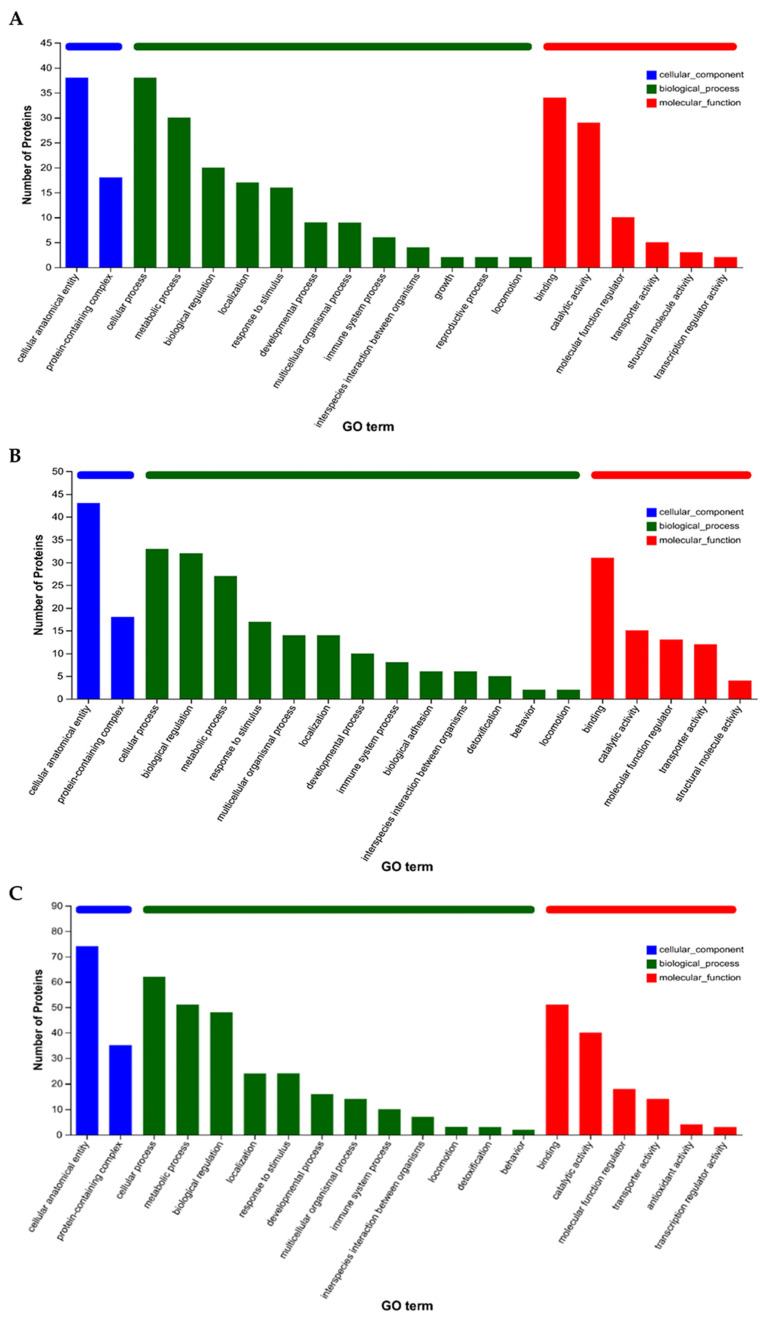

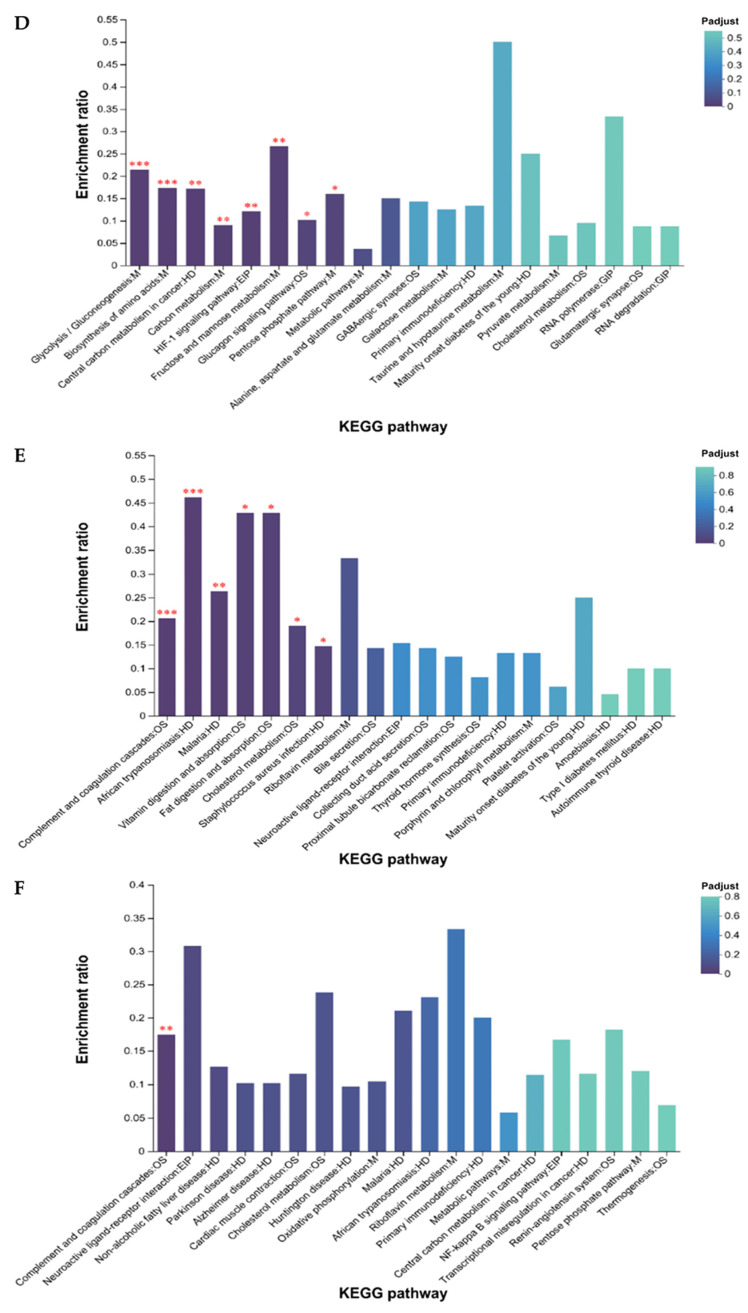
GO and KEGG enrichment analyses of DEPs. (**A**) Numbers of genes falling within enriched GO terms pre- and post-exercise. (**B**) Numbers of genes falling within enriched GO terms post-exercise and 24 h post-exercise. (**C**) Numbers of genes falling within enriched GO terms pre- and 24 h post-exercise. (**D**) Plot of KEGG enrichment analysis comparing pre- and post-exercise. *** denotes *p*-adjust < 0.001, ** denotes *p*-adjust < 0.01, and * denotes *p*-adjust < 0.05. (**E**) Plot of KEGG enrichment analysis comparing post-exercise and 24 h post-exercise. *** denotes *p*-adjust < 0.001, ** denotes *p*-adjust < 0.01, and * denotes *p*-adjust < 0.05. (**F**) Plot of KEGG enrichment analysis comparing pre- and 24 h post-exercise. ** denotes *p*-adjust < 0.01.

Gene Ontology (GO) functional annotation was conducted on the differentially expressed proteins (DEPs). When comparing the pre-exercise state with the post-exercise state (Figure 6A), the DEPs were significantly enriched for terms such as cellular anatomical entity, cellular process, binding, metabolic process, and catalytic activity. In the comparison between the post-exercise state and the state 24 h post-exercise (Figure 6B), the DEPs were notably enriched for terms related to cellular anatomical entity function, cellular process, biological regulation, binding, and metabolic process. For the comparison between the pre-exercise state and the state 24 h post-exercise (Figure 6C), the DEPs were significantly enriched for terms including cellular anatomical entity, cellular process, and metabolic process.

Kyoto Encyclopedia of Genes and Genomes (KEGG) enrichment analysis was performed on the DEPs. In the comparison between the pre-exercise and post-exercise states (Figure 6D), the DEPs were significantly enriched for signaling pathways such as glycolysis/gluconeogenesis, the biosynthesis of amino acids, the HIF-1 signaling pathway, fructose and mannose metabolism, the glucagon signaling pathway, and the pentose phosphate pathway. When comparing the post-exercise state with the state 24 h post-exercise (Figure 6E), the DEPs were significantly enriched for pathways including complement and coagulation cascades, vitamin digestion and absorption, fat digestion and absorption, and cholesterol metabolism. In the comparison between the pre-exercise state and the state 24 h post-exercise (Figure 6F), the results indicated that only one signaling pathway, namely the complement and coagulation cascade pathway, was enriched.

#### 3.2.4. Protein–Protein Interaction Network Analysis of DEPs

**Figure 7:**
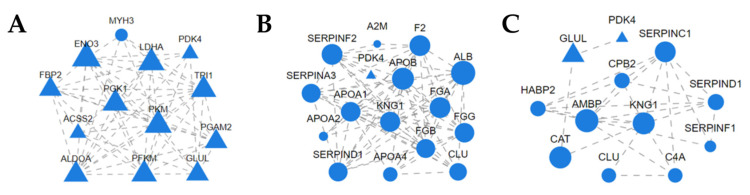
(**A**) Protein interaction network analysis pre- and post-exercise. Nodes represent proteins, with triangles indicating upregulated proteins and circles indicating downregulated proteins. Edges represent interactions between two proteins. The size of a node is proportional to its connectivity; that is, the more edges are connected to this node, the greater its connectivity, and the larger the node, indicating that the gene of this node has greater importance in the network. (**B**) Protein interaction network analysis comparing immediately post-exercise and 24 h post-exercise conditions. (**C**) Protein interaction network analysis comparing pre-exercise and 24 h post-exercise conditions.

In the comparative analysis of protein—protein interaction networks pre- and post- exercise presented in Figure 7A, initially, the interaction network analysis was conducted on all differential proteins that could be connected. Then, based on whether they appeared in pathways and their connectivity degrees, a screening procedure was carried out. We retained the proteins that appeared in pathways and had high connectivity degrees, which are displayed in the figure. A total of 37 nodes and 142 edges were identified. Through screening, proteins with higher relevance are displayed in the figure. Among them, significantly upregulated proteins include LDHA, ENO3, TPI1, FBP2, PGK1, PKM, PGAM2, ACSS2, ALDOA, PFKM, GLUL, and PDK4, whereas the significantly downregulated protein is MYH3. In the comparative analysis of protein–protein interaction networks immediately after exercise and 24 h post-exercise shown in Figure 7B, a total of 55 nodes and 234 edges were identified. The figure displays proteins with higher relevance, among which PDK4 is significantly upregulated; and significantly downregulated proteins include A2M, F2, ALB, SERPINF2, SERPIND1, SERPINA3, APOB, APOA1, APOA2, APOA4, KNG1, FGA, FGB, FGG, and CLU. Finally, in the comparative analysis of protein–protein interaction networks before exercise and 24 h post-exercise depicted in Figure 7C, a total of 86 nodes and 300 edges were identified. The figure showcases proteins with higher relevance, with GLUL and PDK4 being significantly upregulated; and significantly downregulated proteins include CPB2, SERPINC1, HABP2, AMBP, KNG1, SERPIND1, SERPINF1, CAT, CLU, and C4A. For a more comprehensive understanding of the interaction network results, readers can refer to Supplementary Table S5, which contains the node attribute table of the more complete interaction network.

### 3.3. Integrated Analysis of Muscle Transcriptome and Proteome in Mongolian Horses Pre- and Post-Exercise

#### 3.3.1. Integrated Analysis of DEGs and DEPs

**Figure 8:**
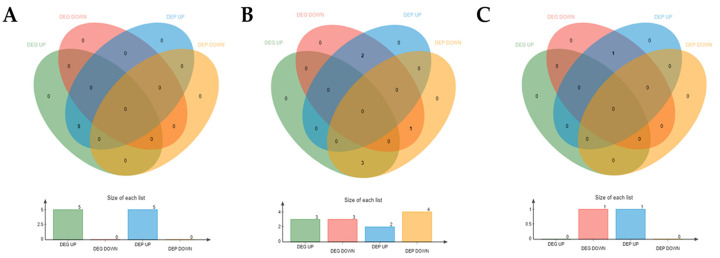
Venn diagram showing differential expression of correlated data. (**A**) Venn diagram showing differential expression of genes and proteins before versus after exercise. (**B**) Venn diagram showing differential expression of genes and proteins immediately after exercise versus 24 h post-exercise. (**C**) Venn diagram showing differential expression of genes and proteins before exercise versus 24 h post-exercise.

To further analyze the key differentially expressed genes (DEGs) and differentially expressed proteins (DEPs) that may influence the internal mechanisms of the change in Mongolian horses pre- and post-exercise, we conducted a joint analysis of RNA-Seq and proteomics data for DEGs and DEPs. When a gene and its corresponding protein are simultaneously expressed, they can be considered correlated. The correlative analysis of transcriptome and proteome data pre- and post-exercise revealed five commonly upregulated DEGs and DEPs: ENO3, PDK4, PGK1, LDHA, and PKM (Figure 8A). The correlative analysis comparing immediately post-exercise and 24 h post-exercise showed that CES1 was commonly downregulated among both DEGs and DEPs. Among the differences, PDK4 and ASB4 were downregulated in DEGs but upregulated in DEPs, while SERPINA3, CLU, and A2M were upregulated in DEGs but downregulated in DEPs (Figure 8B). The correlative analysis comparing pre-exercise and 24 h post-exercise indicated that GLUL was downregulated in DEGs but upregulated in DEPs (Figure 8C).

#### 3.3.2. Cluster Analysis of Expression Levels in Correlated Data

**Figure 9:**
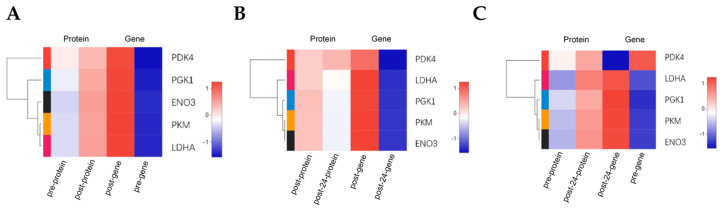
Heat map of correlated data expression. (**A**) Heat map comparing gene and protein expression levels before versus after exercise. (**B**) Heat map comparing gene and protein expression levels immediately after exercise versus 24 h post-exercise. (**C**) Heat map comparing gene and protein expression levels before exercise versus 24 h post-exercise.

The clustering heatmap based on the correlated data indicates that Mongolian horses exhibit significant differences at both the protein and gene expression levels after a 20 km exercise (Figure 9).

#### 3.3.3. GO and KEGG Enrichment Analysis of DEGs and DEPs

To comprehensively understand the functional and pathway-level changes associated with exercise and recovery in Mongolian horses, we conducted GO functional annotation and KEGG pathway enrichment analysis on a comprehensive set of differentially expressed genes (DEGs) and differentially expressed proteins (DEPs) identified across different time points (pre-exercise, post-exercise, and 24 h post-exercise).

**Figure 10:**
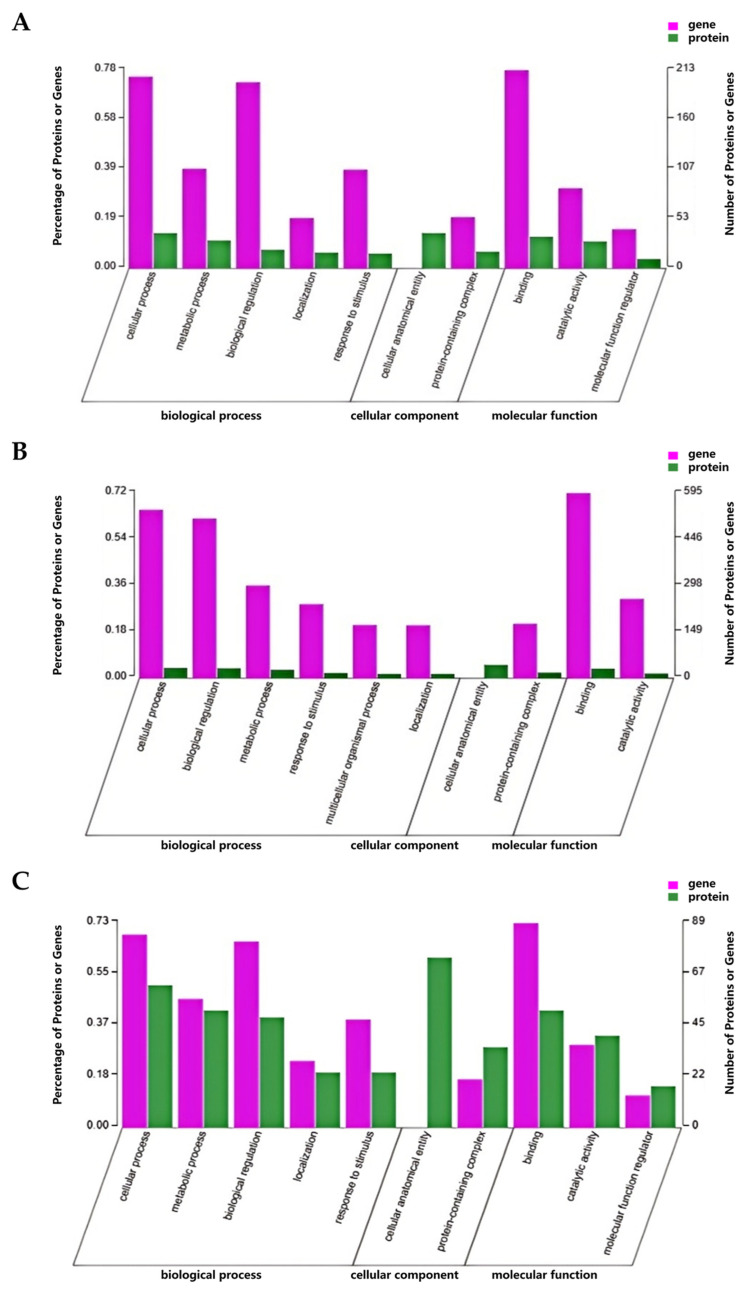

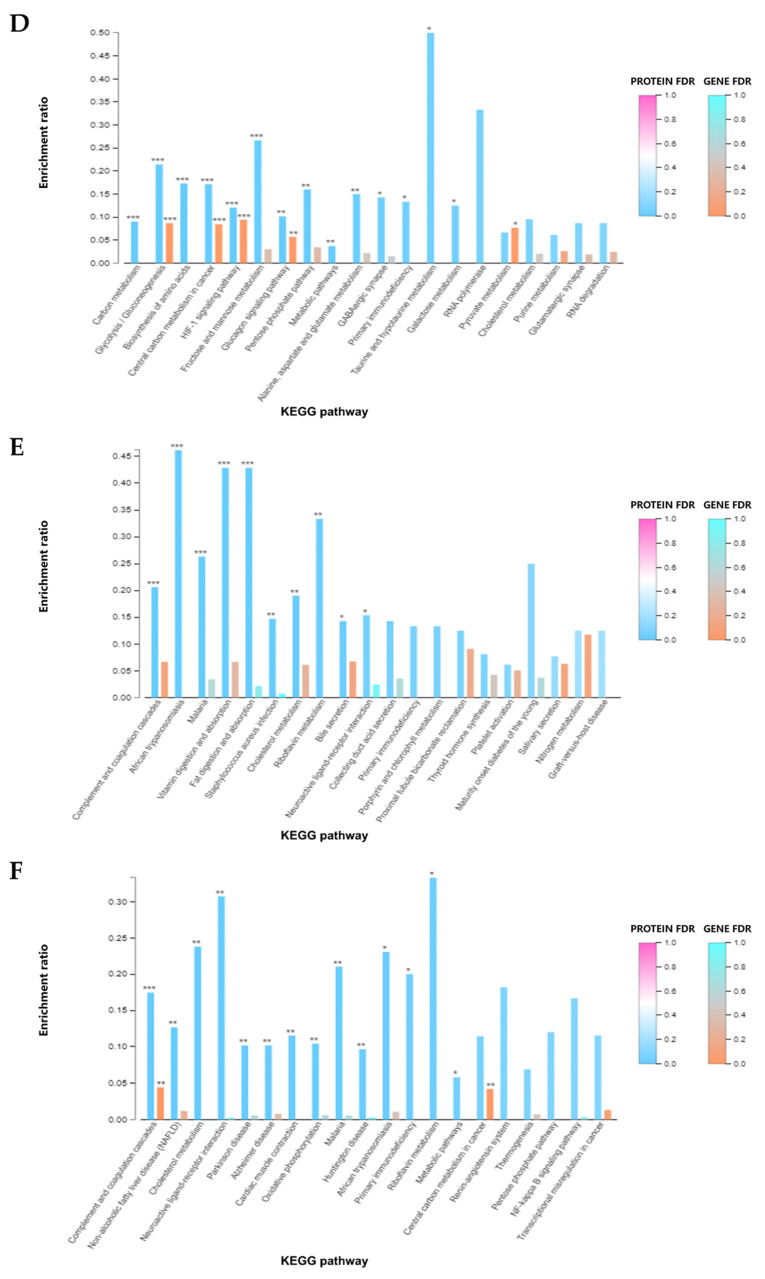
GO and KEGG enrichment analyses of DEGs and DEPs. (**A**) Statistics for GO classification pre- and post-exercise. (**B**) Statistics for GO classification immediately post-exercise and 24 h post-exercise. (**C**) Statistics for GO classification pre- and 24 h post-exercise. (**D**) Plot of KEGG enrichment analysis comparing pre- and post-exercise. *** denotes FDR < 0.001, ** denotes FDR < 0.01, and * denotes FDR < 0.05. (**E**) Plot of KEGG enrichment analysis comparing post-exercise and 24 h post-exercise. *** denotes FDR < 0.001, ** denotes FDR < 0.01, and * denotes FDR < 0.05. (**F**) Plot of KEGG enrichment analysis comparing pre- and 24 h post-exercise. *** denotes FDR < 0.001, ** denotes FDR < 0.01, and * denotes FDR < 0.05.

For the GO functional annotation, we integrated all DEGs and DEPs and analyzed their functional enrichment. In the comparison between pre-exercise and post-exercise (Figure 10A), significant enrichment was observed for terms related to binding function, cellular process function, and biological regulation. Similarly, in the comparison between post-exercise and 24 h post-exercise (Figure 10B), notable enrichment was found for terms such as binding function, cellular process, and biological regulation. When comparing pre-exercise with 24 h post-exercise (Figure 10C), significant enrichment was detected for terms including binding function, cellular process function, and metabolic process. These findings suggest that exercise and recovery processes involve a wide range of biological functions and processes.

For the KEGG pathway enrichment analysis, we also considered all identified DEGs and DEPs together. In the pre-exercise vs. post-exercise comparison (Figure 10D), significant enrichment was identified for pathways such as glycolysis/gluconeogenesis, the HIF-1 signaling pathway, and the glucagon signaling pathway, indicating the activation of energy metabolism and stress response pathways during exercise. In the post-exercise vs. 24 h post-exercise comparison (Figure 10E), although no commonly significantly enriched signaling pathways were observed, it suggests that the recovery process may involve more subtle and specific pathway regulations. Lastly, for the pre-exercise vs. 24 h post-exercise comparison (Figure 10F), the results indicated that the complement and coagulation cascade signaling pathway was significantly enriched, suggesting a potential role of this pathway in the long-term adaptation to exercise.

## 4. Discussion

### 4.1. Transcriptome Changes in Muscles of Mongolian Horses Pre- and Post-Exercise

Prolonged high-intensity exercise may suppress immune function and induce inflammation, and its pathophysiological conditions may be associated with the dysregulation of cellular immune modulation [24]. During intense exercise, protein synthesis in skeletal muscle is inhibited [25,26]. This study found that the activation of the FOXO signaling pathway was significantly upregulated after exercise, which may be mainly related to two factors. On the one hand, engaging in moderate or high-intensity physical activity exacerbates the production of reactive oxygen species (ROS), and the FOXO signaling pathway may be activated due to the increased ROS production. Enhanced FOXO signaling promotes FOXO nuclear translocation and enhances the transcription of FOXO target genes, such as *MAFbx* and *MuRF-1*, which are directly involved in muscle catabolism [27]. On the other hand, the FOXO transcription factor family influences multiple cellular activities by modulating gene expression. Under the stimulation of insulin or growth factors, FOXO proteins are phosphorylated by PI3K-Akt/PKB and translocate from the nucleus to the cytoplasm, reducing their expression. Under oxidative and nutritional stress, JNK and AMPK are activated, which in turn phosphorylate and activate FOXO transcription factors [28]. Additionally, FOXO transcription factors are regulated by various post-translational modification mechanisms, and they inhibit cell proliferation by regulating the expression of cell cycle-related genes [29,30]. In this study, *P21* and *P27* were significantly upregulated after intense exercise and significantly downregulated 24 h later, indicating that intense exercise promoted oxidative phosphorylation levels, as well as cell proliferation and cycle progression in Mongolian horses, which returned to initial levels after 24 h.

The changes in the PI3K-AKT signaling pathway after exercise are also noteworthy. In this study, *PI3K* was significantly upregulated after exercise, and Mongolian horses enhanced their cellular antioxidant capacity through this upregulation. *PI3K* returned to initial levels after 24 h. The PI3K-AKT signaling pathway promotes various physiological processes in cells, relying on two key genes, *PI3K* and *Akt/PKB* [31,32,33]. *AKT* regulates apoptosis and glucose metabolism by phosphorylating *GSK3* isoforms [34,35]. In this study, *GSK3* was significantly upregulated in Mongolian horses after exercise, confirming the regulation of glycolysis/gluconeogenesis through the AKT pathway.

In this study, hypoxia-related factors also showed significant changes after exercise. The hypoxia-inducible factor HIF-1 promotes the shift in energy metabolism from oxidative phosphorylation to glycolysis, reducing intracellular ROS production. For example, it inhibits mitochondrial respiration and electron transport chain activity and activates the transcription of genes encoding glucose transporters and glycolytic enzymes, promoting the expression of the apoptotic protein BNIP3 [36,37]. In this study, the *BNIP3* gene showed significant changes after exercise and returned to normal after 24 h, consistent with the function of the HIF-1 pathway. In animal cells, HIF and its regulators regulate the perception and response to oxygen. HIF-1 activates genes encoding proteins involved in the hypoxic homeostatic response and induces the expression of proteins related to glucose metabolism, cell proliferation, and angiogenesis [38,39]. In this study, *LDHA* was upregulated in the HIF-1 signaling pathway after intense exercise and remained elevated 24 h later. LDHA is a key glycolytic enzyme, and excessive lactate can cause extracellular acidosis, promoting invasion, angiogenesis, and metastasis and affecting the immune response [40].

There is an interaction between hypoxia and p53 [41]. Hypoxia induces p53 protein accumulation, and p53 can inhibit HIF1-α expression by promoting MDM2-mediated ubiquitination and proteasomal degradation pathways. HIF1-α can also block p53-mediated degradation and bind to hepatocyte nuclear factor 4α (HNF-4α) to activate the expression of the erythropoietin (*Epo*) gene [42,43]. P53 induces repair or apoptosis in DNA-damaged cells. Cellular metabolism is a process controlled by p53, and the effects of p53 on the same metabolic process may vary in different cell types [44,45]. In this study, the genes *P21* and *Gadd45* enriched in the p53 signaling pathway showed significant changes after exercise and returned to normal after 24 h, consistent with the changes in the *Gadd45* gene after being affected by ROS.

### 4.2. Proteome Changes in Muscles of Mongolian Horses Pre- and Post-Exercise

During exercise, Mongolian horses experience increased energy consumption and accelerated metabolic rates, and changes in the internal environment lead to exercise-induced fatigue [46]. During intense exercise, cells produce ATP through mitochondrial respiration to supply energy to the body [47]. Energy metabolism involves the production of energy (ATP) and small-molecule metabolites from nutrients such as glucose and fatty acids through anaerobic glycolysis and aerobic respiration [48]. This study showed that after intense exercise, signaling pathways such as amino acid biosynthesis and fructose and mannose metabolism were significantly enriched in the muscle proteome of Mongolian horses. Key proteins, including PDK4, PFKM, LDHA, PGAM, and ALDOA, were upregulated, thereby promoting an increase in glycolysis levels.

Among them, PFKM, as an important regulatory enzyme in glycolysis, can inhibit doxorubicin-induced apoptosis and reduce glycolysis and oxidative phosphorylation through overexpression. It can also regulate doxorubicin-mediated cell viability and apoptosis [49]. LDH is a key enzyme in glycolysis, catalyzing the conversion of pyruvate to lactate. Its upregulation after exercise means that intense exercise increases lactate production and accelerates glycolytic reactions. PGAM translocates from the mitochondria to the cytoplasm with an increase in intracellular ROS levels, and its mRNA and total protein expression first decrease and then increase. Moderate ROS can induce an increase in its expression [50,51]. ALDOA is a key enzyme in glycolysis and gluconeogenesis and an important driving gene for the hypoxic growth of liver cancer cells [52]. Its upregulation can regulate the NLRP3 inflammasome by sensing changes in glycolytic flux, inhibit the body’s inflammatory response, and also control the AMPK–mitophagy signaling pathway to maintain mitochondrial damage [53]. PDK4 was significantly upregulated after exercise and remained elevated 24 h later. It is a key regulatory protein targeting mitochondrial decarboxylation and regulates mitochondrial fatty acid oxidation and ATP production by phosphorylating and inhibiting pyruvate dehydrogenase activity. It plays an important role in the regulation of glucose and fatty acid metabolism. Its expression is regulated by hyperglycemia and fasting conditions, affects the choice of glucose metabolic pathways, and is closely related to the balance of energy metabolism. Its upregulation marks an overall metabolic shift, increasing the utilization of fatty acids as an energy source and serving as a sensitive marker of enhanced fatty acid oxidation [54,55]. During intense exercise, the body’s energy demand surges, leading to a decline in muscle cell phosphocreatine levels. Muscle glycogen is converted, resulting in lactate accumulation, and when oxygen supply is insufficient, the body switches to anaerobic metabolism. Recovery from such exercise involves replenishing glycogen reserves, and all involved processes require energy. These data suggest that during and after intense exercise, the body undergoes significant metabolic adjustments to meet energy demands and initiate recovery.

Intense exercise also induces a series of stress responses and metabolic adaptations. Mongolian horses can undergo a certain degree of self-recovery after running 20 km. In terms of lipid metabolism, apolipoproteins are synthesized in the intestine and liver and transport lipids. APOA1 is the main protein component for transporting cholesterol on HDL [56], and APOA4 promotes lipid transport and metabolism. In this study, KEGG pathway enrichment analysis of the proteome found that APOB, APOA1, and APOA4 in pathways such as vitamin digestion and absorption were significantly downregulated after exercise and 24 h later, indicating that exercise can regulate apolipoprotein-mediated immune regulation in the body through these pathways. GLUL catalyzes the conversion of glutamate and ammonia to glutamine, which is important for various biological processes [57]. Its significant upregulation after exercise and persistence 24 h later indicate that intense exercise promotes cell proliferation to counteract damage. AMBP is expressed in various tissues and regulates multiple physiological functions, with strong antioxidant activity [58]. In this study, it was significantly downregulated 24 h after exercise compared to before exercise. It is involved in liver lipid metabolism and protein degradation and is crucial for maintaining the body’s energy balance and nutrient transport. In terms of antioxidant function, CAT is an oxidative scavenging enzyme for H_2_O_2_, which can eliminate intracellular hydrogen peroxide and protect cell function [59]. Proteomic analysis in this study showed that CAT was significantly downregulated after exercise and remained downregulated 24 h later. Long-term endurance exercise is prone to cause oxidative stress. Our data, along with findings from previous studies [60,61], suggest that the decline in antioxidant indicators such as SOD, CAT, and GSH-Px after exercise may be due to an increase in the number of reactive oxygen species in the body, potentially leading to oxidative damage and cellular dysfunction.

After running 20 km, Mongolian horses generate an immune response and enter a stage of self-replenishment and recovery. Twenty-four h after exercise, the complement and coagulation cascade signaling pathways were significantly enriched. The complement factors CLU and C4A, as well as coagulation-related factors such as F2, FGA, etc., were downregulated and remained so until 24 h later. CLU is a complement signal inhibitor, which was significantly downregulated after exercise and remained so 24 h later. It can exert anti-inflammatory effects in both acute and chronic inflammation [62,63]. The complement factor C4A was downregulated 24 h after exercise. Endurance exercise induces the activation of the alternative pathway of the complement system, mediating the involvement of complement factors in post-exercise recovery [64,65]. Coagulation factor II (F2) forms activated thrombin, which plays an important role in thrombosis and hemostasis and is also involved in cell proliferation. FGA, FGB, and FGG are the three main subunits of fibrinogen and are crucial in blood coagulation and hemostasis. The serine protease inhibitor (Serpin) superfamily is involved in various life activities. SERPIND1 is associated with multiple diseases. SERPINF2 mainly acts as a plasmin inhibitor. SERPINA3 inhibits digestive enzyme activity, maintains the balance of protein digestion in the intestine, and is also related to various biological processes. Fibrinogen limits blood coagulation by reducing thrombin activity. Twenty-four hours after exercise, during the recovery period, coagulation-related proteins and the complement factor CLU remained downregulated, which may lead to coagulation disorders and a reduced immune response speed in Mongolian horses that have undergone excessive exercise.

### 4.3. Integrated Analysis of Muscle Transcriptome and Proteome in Mongolian Horses Pre- and Post-Exercise

Multi-omics data analysis (such as the combination of RNA-Seq and proteomics) can provide higher accuracy compared to single-omics analysis and has been widely applied in the study of biological systems [66]. By integrating RNA-Seq and proteomic data, the complex relationship between gene expression and protein regulation can be revealed, further understanding the adaptive mechanisms of Mongolian horses after intense exercise. Studies have shown that the expression levels of genes and proteins are not always correlated [67]. In this study, the correlation between DEGs and DEPs was low, which may be caused by post-transcriptional and post-translational regulatory mechanisms [68,69]. Through joint analysis, key genes and proteins were identified, providing new perspectives for improving the exercise performance of Mongolian horses and promoting the development of the modern horse industry.

In terms of energy metabolism, under hypoxic conditions, the production of reactive oxygen species (ROS) is known to increase, which can induce HIF-1α to reduce ROS levels through various mechanisms, such as improving the efficiency of complex IV, promoting the conversion of pyruvate to lactate, and triggering selective mitochondrial autophagy [70]. While our study did not directly measure ROS levels in Mongolian horses after exercise, previous research suggests that ROS production can increase following exercise [71,72]. Based on these findings and the context of our study, it is plausible that the HIF-1 signaling pathway may upregulate genes such as *PI3K*, *LDHA*, and *PFK2* to enhance cellular antioxidant capacity and glycolytic function in Mongolian horses after exercise. Glycolysis-related genes, such as *LDHA*, *PFK2*, *PFKM*, and *ALDOA*, were found to be upregulated in our study, promoting cell energy acquisition and proliferation. Additionally, proteins related to the glucagon signaling pathway, such as FBP2 and LDHA, were also upregulated, potentially inhibiting apoptosis induced by oxidative stress and promoting cell glycolysis and oxidative phosphorylation.

The complement system and the coagulation cascade play key roles in maintaining the body’s immune health [73]. After intense exercise, the complement signal inhibitor CLU is significantly downregulated, and the complement factor C4A is also downregulated 24 h after exercise, which may affect post-exercise recovery. Coagulation-related factors, such as F2, SERPIND1, SERPINF2, SERPINA3, FGA, FGB, and FGG, are also downregulated, leading to coagulation dysfunction in the body, which is not conducive to post-exercise recovery and can even lead to thrombosis in severe cases. These changes indicate that intense exercise has a significant impact on the complement and coagulation systems. However, in future studies, further research on the molecular mechanisms and potential interventions is needed.

## 5. Conclusions

In this study, our integrated transcriptomic–proteomic analysis of Mongolian horses’ muscles during and after a 20 km run highlights key genes and proteins (e.g., PI3K, LDHA, and PFK2) involved in adaptive pathways like HIF-1 signaling and glycolysis, revealing insights into their endurance mechanisms with implications for performance enhancement and exercise biology.

## Data Availability

The original contributions presented in the study are included in the article/Supplementary Materials, further inquiries can be directed to the corresponding author.

## Supplementary Materials

The following supporting information can be downloaded at https://www.mdpi.com/article/10.3390/ani15131981/s1. Table S1. Reverse transcription reaction system; Table S2. Primer information. Table S3: Reagent composition; Table S4. Amplification procedure. Table S5: Node attribute table of the network.
